# Improving the Performance of a Post-Buckled Beam Harvester under Combined External and Parametrical Slow Excitations

**DOI:** 10.3390/mi14061238

**Published:** 2023-06-12

**Authors:** Yue Zhou, Jinchao Cui, Wenan Jiang

**Affiliations:** 1 Educational Technology and Information Center, Guangdong Medical University, Dongguan 523808, China; zhouyue12152023@163.com; 2 School of Biomedical Engineering, Guangdong Medical University, Dongguan 523808, China; 3 Faculty of Civil Engineering and Mechanics, Jiangsu University, Zhenjiang 212013, China

**Keywords:** energy harvesting, slow excitations, commensurate excitation frequencies, buckled beam

## Abstract

In this paper, we consider novel bursting energy harvesting under combined external and parametrical slow excitations, and a harvester is realized by employing an externally and parametrically excited post-buckled beam. Based on the method of fast–slow dynamics analysis, multiple-frequency oscillation, with two slow commensurate excitation frequencies, is used to observe complex bursting patterns, the behaviors of the bursting response are presented, and some novel one-parameter bifurcation patterns are observed. Furthermore, the bursting harvesting performances of the single and the two slow commensurate excitation frequencies are compared, and it was found that the two slow commensurate excitation frequencies can be used to improve the harvesting voltage.

## 1. Introduction

Non-linear responses of buckled beams have been studied by many research groups, which have all contributed to analyzing the non-linear behavior of buckled beams possessing post-buckling and internal resonances [1,2,3,4]. Masana and Daqaq pioneered the use of a buckled beam as an energy harvester for broadband harvesting [5], and different potential characteristics are discussed [6]. Jiang et al. [7] designed a buckled beam harvester with an oscillator and proposed internal resonance to improve the harvesting bandwidth. Panyam et al. [8] investigated the dynamical behavior and harvesting performance of a parametrically excited buckled beam harvester. As of today, bursting oscillation for externally and parametrically excited buckled beam energy harvesters is still open for study. Bursting oscillation as a typical representative of fast–slow dynamical behaviors, and it is frequently encountered in various fields of science and engineering. Scavenging the vibratory energy of bursting oscillation to run low-power-consumption electron devices is a popular topic. Therefore, it is urgent to study the bursting oscillations as well as the bursting mechanism of energy harvesting systems under combined external and parametrical slow excitations.

It is well known that bursting oscillation has attracted widespread attention due to its explosive dynamic pattern from multiple timescale systems. Recently, bursting dynamics have been extensively emphasized in terms of the mechanical structure [9,10], memristive oscillator [11,12], chemical reaction [13,14], nonlinear system [15,16], and sandwich conical panel [17]. In addition, the complex bursting patterns in a parametrically and externally excited nonlinear dynamical system have been widely investigated in various systems. For example, Han et al. presented a general method for analyzing mixed-mode oscillations in the parametrically and externally excited Duffing equation [18] and Rayleigh-Duffing equation [19,20]. Ma et al. [21] considered compound bursting in a modified van der Pol–Duffing circuit system with slow-varying periodic excitation. Wen et al. [22] reported several compound bursting patterns in a memristor-based Shimizu–Morioka system. Wei et al. reported compound bursting dynamics in a parametrically and externally excited quintic nonlinear Rayleigh–Duffing system [23] and Jerk circuit system [24]. Yu et al. [25] performed analytical investigations on symmetric jump phenomena reflecting multi-timescale dynamics in a nonlinear shape memory alloy oscillator with parametric and external cosinoidal excitations. Chen et al. [26] proposed the parametrically excited vibrations and mode transitions of a nonlinear damped triple-well oscillator, revealing the multiple timescale structure of an oscillator with resonant frequency. Zhang et al. [27] explored novel multiple-frequency bursting of a shape memory oscillator under parametrical and external excitation. However, most of the works focus on nonlinear dynamic behaviors, and there is little publication on vibration utilization. Recently, bursting energy harvesting has become a new topic [28,29,30,31,32,33], but most publications focus on external excitation; bursting oscillation of externally and parametrically excited energy harvesting is not reported. Therefore, to resolve this issue, bursting oscillations of an externally and parametrically excited post-buckled beam harvester are implemented.

To fill this void in the open literature, this article is meant to employ novel bursting oscillation of a parametrically and externally excited post-buckled beam harvester with multiple double-frequency slow excitations. To achieve this aim, a buckled beam harvester under combined parametric and external base excitations was designed. The bursting mechanism of slowly varying frequency was investigated, and it was revealed that the system exhibits rich dynamic behaviors at different frequency ratios. Particularly, the two slow commensurate excitation frequencies can be used to improve the harvesting voltage.

The structure of this paper is as follows. Section 2 gives a problem formulation of an externally and parametrically excited buckled beam harvester. In Section 3, the bursting mechanism and patterns of an external and parametrical vibration are investigated. In Section 4, the bursting harvesting performances of the single- and double-frequency slow excitations are compared. In Section 5, the proposed bursting results are verified by employing the Simulink. Finally, Section 6 concludes the paper.

## 2. Basic Model

**Figure 1:**
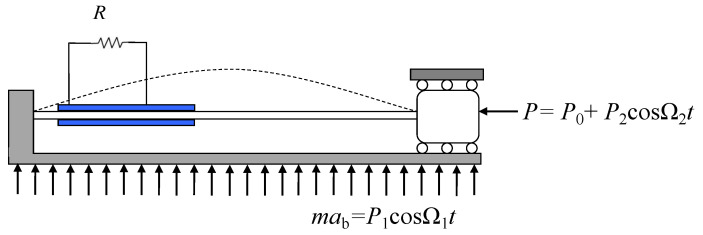
A schematic of an externally and parametrically excited buckled beam harvester.

The physical mechanical model of an externally and parametrically excited buckled beam harvester is depicted in Figure 1, and the correspondingly mathematical model can be performed as

(1) $$m\ddot{q}+c\dot{q}+k\left[1-\alpha \left(P_{0}+P_{2}\cos\left(\Omega_{2}t\right)\right)\right]q+\gamma \left(1-\beta p\right)q^{3}+\theta v=P_{1}\cos\left(\Omega_{1}t\right)$$

(2) $$c_{p}\dot{v}+\frac{v}{r}-\theta \dot{q}=0\;$$

where *m* denotes an effective mass of the buckled beam, *q* is the displacement of a beam, *k* is the linear stiffness of the harvester, *c* defines the structure damping, γ is the nonlinear stiffness coefficient, α is the reciprocal of critical load, β is a constant for brevity, *v* is the output voltage of the harvesting, θ is the electromechanical coupling value, $c_{p}$ is the capacitance, *r* is the resistive load, and $P_{1}\cos\left(\Omega_{1}t\right)$ and $P_{2}\cos\left(\Omega_{2}t\right)$ are external and parametrical excitations, respectively.

For the convenience of subsequent analysis, the mathematical Equations (1) and (2) can be rewritten as the dimensionless form

(3) $$\ddot{x}+\xi \dot{x}-\eta x+\delta x^{3}-f_{2}\cos\omega_{2}tx+\kappa \nu =f_{1}\cos\omega_{1}t$$

(4) $$\dot{\nu }+\chi \nu -\lambda \dot{x}=0$$

## 3. Bursting Mechanism of an Externally and Parametrically Excited Energy Harvester

### 3.1. Bursting Mechanism of a Single-Frequency Slow Excitation

In this section, we analyze the dynamical response of the externally and parametrically excited post-buckled beam harvester and investigate the behavior of slowly varying frequencies, namely, $0<\omega_{1}=\omega_{2}\ll 1$. Equations (3) and (4) then become one with only a single-frequency excitation, and the fast subsystem of differential Equations (3) and (4) is measured as

(5) $$\ddot{x}+\xi \dot{x}-x+x^{3}-f_{2}\sigma x+\kappa \nu =f_{1}\sigma$$

(6) $$\dot{\nu }+\chi \nu -\lambda \dot{x}=0$$

where $\sigma =\cos(\omega_{1}t)$ is a slowly varying constraint parameter. The static equilibrium equation can be performed as

(7) $$-x_{e}+{x_{e}}^{3}-f_{2}\sigma x_{e}=f_{1}\sigma$$

and the equilibrium locations can be conveniently calculated by the numerical method. Furthermore, the stability of equilibrium location can be determined by the Lyapunov theory [28].

**Figure 2:**
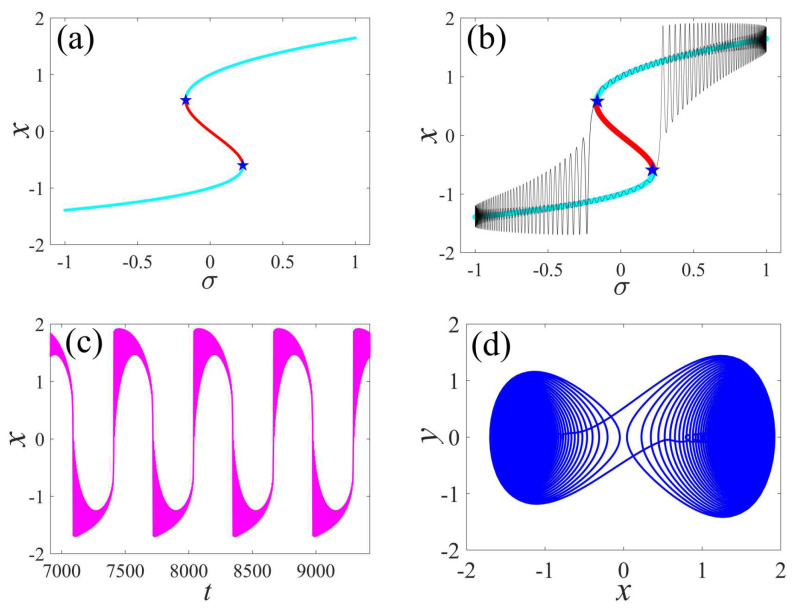
Bursting response of an externally and parametrically excited post-buckled beam harvester: (**a**) bifurcation of equilibrium points, (**b**) transformed phase, (**c**) time series, and (**d**) phase portraits. (The solid cyan lines represent the stable values, the red lines represent the unstable values, the blue pentagram represents the fold bifurcation points, the black lines represent the time series within a cycle, the magenta lines denote time series, and the blue line defines phase portraits).

Next, the bursting mechanism of the externally and parametrically excited post-buckled beam harvester is analyzed. Figure 2 shows the bifurcation of equilibrium points, transformed curves, time series, and phase portraits. As shown in Figure 2a, the curve of the equilibrium points exhibits two-fold bifurcation points, where the solid cyan lines represent the stable values, the red lines represent the unstable values, and the blue pentagram represents the fold bifurcation points. The annotation of the bifurcation graph adopts the form of uniform color, and the legends below are not repeatedly described. Meanwhile, the static equilibrium curve has an equilibrium point when σ is larger than and less than the value of the fold bifurcation point, and it has three solutions when σ lies between the two-fold bifurcations. Figure 2b shows the transformed curves of the equilibrium points, and it can be observed that two jump phenomenons occur at the bifurcation points, which also depend on the path and which exhibit hysteresis behavior. In addition, it can be described that the time series is consistent with the bifurcation of the equilibrium points, which verifies the correctness of the bifurcation curve. In the meantime, the corresponding time series and phase portraits are plotted in Figure 2c,d, respectively.

### 3.2. Bursting Patterns of Two Slow Commensurate Excitation Frequencies

When $\omega_{2}$ is not equal to $\omega_{1}$, there are many different types of frequency values in this case, and the situation becomes very complicated. Interesting bursting patterns may appear in this condition, and even novel bifurcation diagrams are explored. In this section, we check the influence of two slow commensurate excitation frequencies that are bursting. The governing equation can be formatted as

(8) $$\ddot{x}+\xi \dot{x}-x+x^{3}-f_{2}\cos\omega_{2}tx+\kappa \nu =f_{1}\cos\omega_{1}t$$

(9) $$\dot{\nu }+\chi \nu -\lambda \dot{x}=0$$

Here, we assume that the fundamental frequency of the slowly varying excitation $\omega_{0}$ is 0.01. It is interesting to perform $\omega_{2}$ and $\omega_{1}$ as an integer relationship of $\omega_{0}=0.01$. In general, $f_{2}\cos\left(\omega_{2}t\right)$ and $f_{1}\cos\left(\omega_{1}t\right)$ can be expanded as the function of $\omega_{0}=0.01$, namely, $\cos\left(\omega_{2}t\right)=\upsilon_{2n}^{*}\left(\cos\left(\omega_{0}t\right)\right)$ and $\cos\left(\omega_{1}t\right)=\upsilon_{1n}^{*}\left(\cos\left(\omega_{0}t\right)\right)$ yield

(10) $$\upsilon_{n}^{*}\left(\sigma \right)=c_{n}^{0}\sigma^{n}-c_{n}^{2}\sigma^{n-2}\left(1-\sigma^{2}\right)+c_{n}^{4}\sigma^{n-4}{\left(1-\sigma^{2}\right)}^{2}+\cdots +i^{p}c_{n}^{p}\sigma^{n-p}{\left(1-\sigma^{2}\right)}^{\frac{p}{2}}$$

where σ is $\cos(\omega_{0}t)$, *i* is the imaginary unit, $c_{n}^{p}$ is the combinatorial expression, and *p* is the maximum even value that is smaller than *n*. Then, the corresponding fast system of Equations (8) and (9) is remarked as

(11) $$\ddot{x}+\xi \dot{x}-x+x^{3}-f_{2}\upsilon_{2n}^{*}\left(\sigma \right)x+\kappa \nu =f_{1}\upsilon_{1n}^{*}\left(\sigma \right)$$

(12) $$\dot{\nu }+\chi \nu -\lambda \dot{x}=0$$

**Figure 3:**
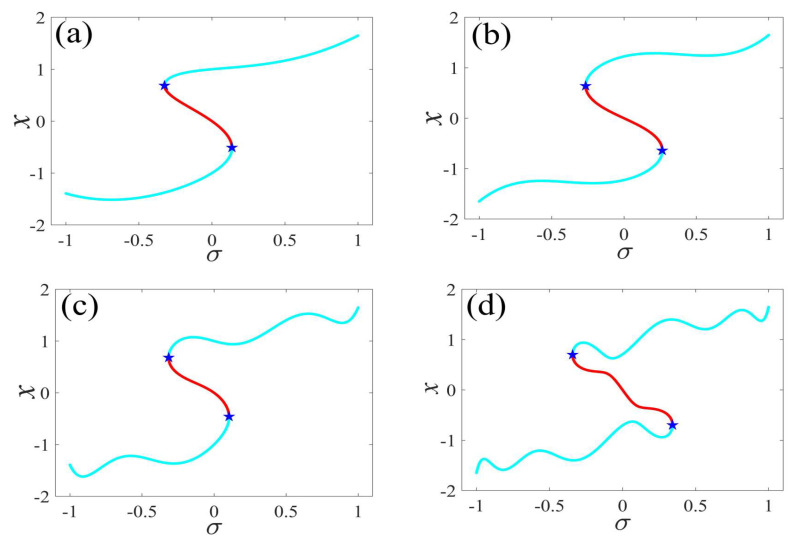
One-parameter bifurcation patterns of $\omega_{1}=0.01$ for (**a**) $\omega_{2}=0.01$, (**b**) $\omega_{2}=0.04$, (**c**) $\omega_{2}=0.07$, and (**d**) $\omega_{2}=0.10$. (The solid cyan lines represent the stable values, the red lines represent the unstable values, the blue pentagram represents the fold bifurcation points).

**Figure 4:**
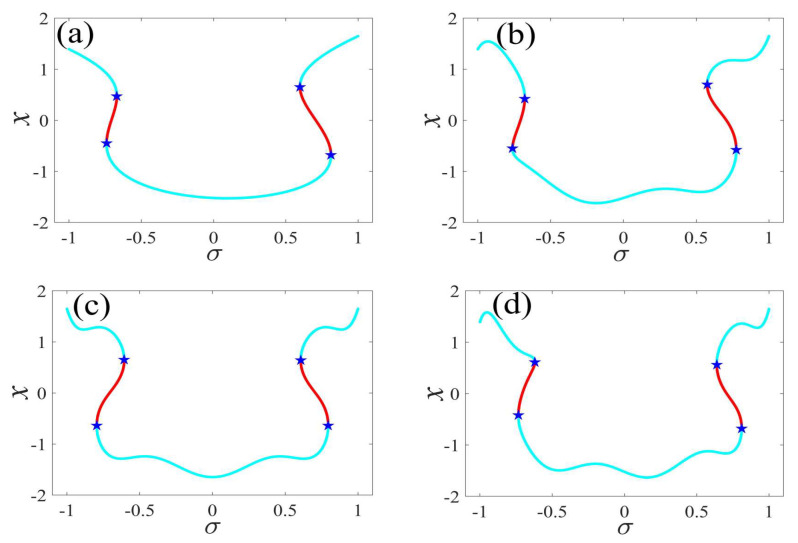
One-parameter bifurcation patterns of $\omega_{1}=0.02$ for (**a**) $\omega_{2}=0.01$, (**b**) $\omega_{2}=0.07$, (**c**) $\omega_{2}=0.08$, and (**d**) $\omega_{2}=0.09$. (The solid cyan lines represent the stable values, the red lines represent the unstable values, the blue pentagram represents the fold bifurcation points).

**Figure 5:**
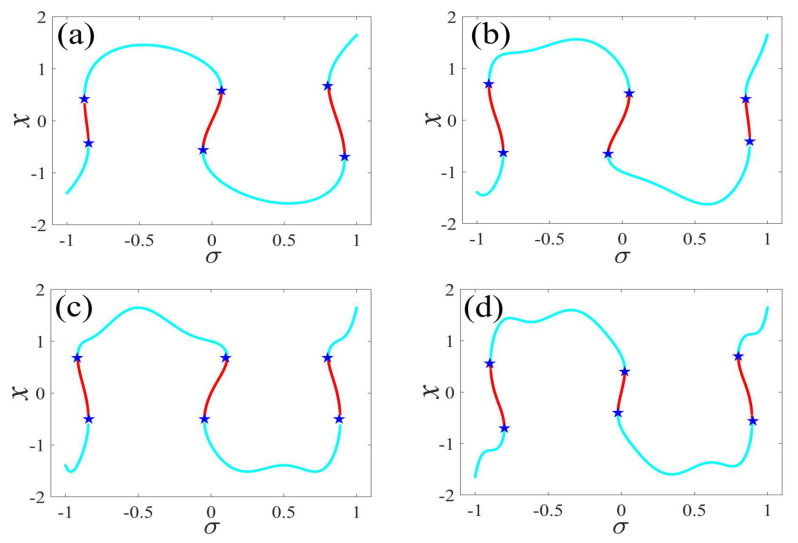
One-parameter bifurcation patterns of $\omega_{1}=0.03$ for (**a**) $\omega_{2}=0.01$, (**b**) $\omega_{2}=0.07$, (**c**) $\omega_{2}=0.09$, and (**d**) $\omega_{2}=0.10$. (The solid cyan lines represent the stable values, the red lines represent the unstable values, the blue pentagram represents the fold bifurcation points).

**Figure 6:**
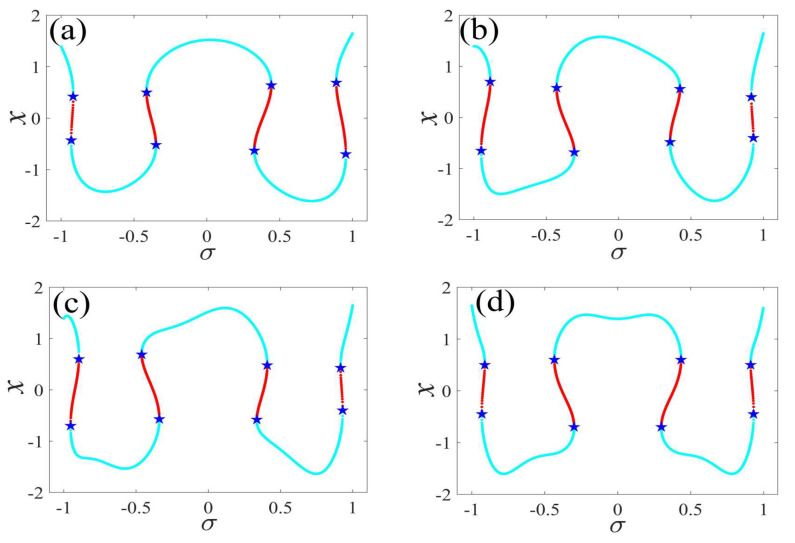
One-parameter bifurcation patterns of $\omega_{1}=0.04$ for (**a**) $\omega_{2}=0.01$, (**b**) $\omega_{2}=0.07$, (**c**) $\omega_{2}=0.09$, and (**d**) $\omega_{2}=0.10$. (The solid cyan lines represent the stable values, the red lines represent the unstable values, the blue pentagram represents the fold bifurcation points).

**Figure 7:**
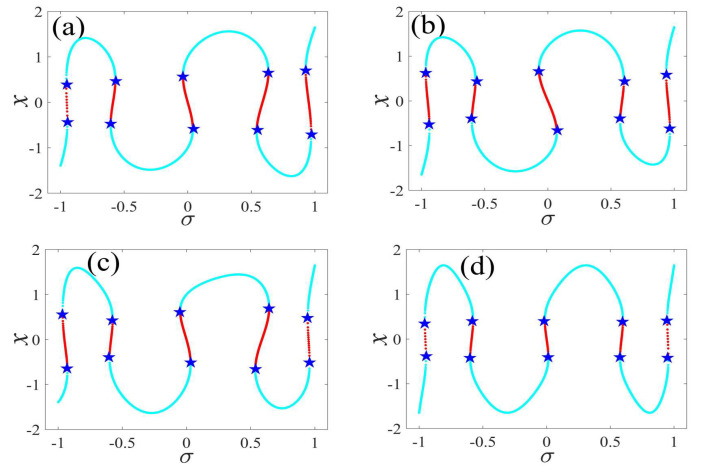
One-parameter bifurcation patterns of $\omega_{1}=0.05$ for (**a**) $\omega_{2}=0.01$, (**b**) $\omega_{2}=0.04$, (**c**) $\omega_{2}=0.07$, and (**d**) $\omega_{2}=0.10$. (The solid cyan lines represent the stable values, the red lines represent the unstable values, the blue pentagram represents the fold bifurcation points).

**Figure 8:**
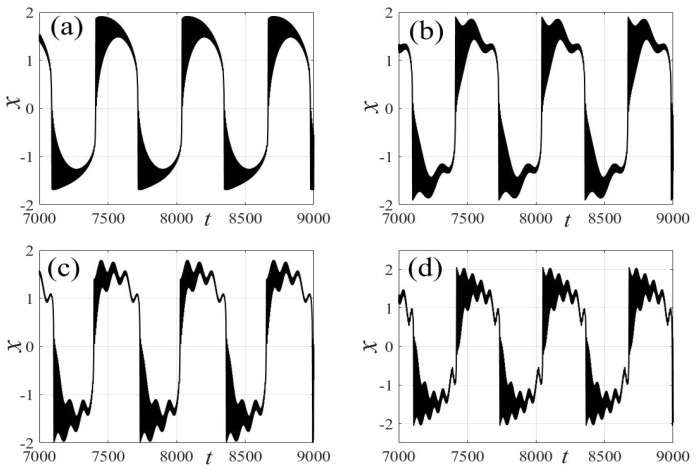
Bursting patterns of displacement with $\omega_{1}=0.01$ for (**a**) $\omega_{2}=0.01$, (**b**) $\omega_{2}=0.04$, (**c**) $\omega_{2}=0.07$, and (**d**) $\omega_{2}=0.10$. (The black lines represent time series).

**Figure 9:**
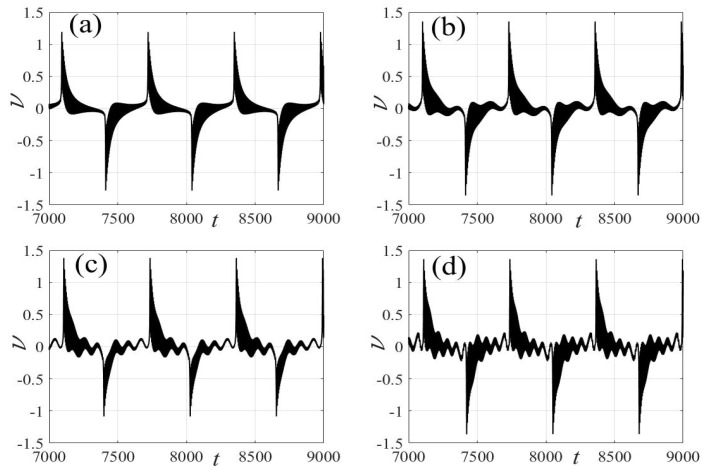
Bursting patterns of voltage with $\omega_{1}=0.01$ for (**a**) $\omega_{2}=0.01$, (**b**) $\omega_{2}=0.04$, (**c**) $\omega_{2}=0.07$, and (**d**) $\omega_{2}=0.10$. (The black lines represent time series).

In the following discussion, the mechanism of bursting will be parsed to reflect the influence of multiple frequencies. Letting ζ=0.02, $f_{1}=2$, $f_{2}=0.5$, some bifurcation curves of some typical frequency ratios are performed in Figure 3, Figure 4, Figure 5, Figure 6 and Figure 7. From these figures, we can see that as the growth of the frequency $\omega_{2}$ increases for a fixed $\omega_{1}$, the number of bends of the equilibrium curves increases. In addition, as the frequency $\omega_{1}$ grows for a fixed $\omega_{2}$, the number of bifurcation points in the equilibrium curves increases. Meanwhile, the number of unstable solution intervals is equal to the frequency ratio $\omega_{1}$ to $\omega_{0}$, and the number of folded bifurcation points is twice the frequency ratio. In addition, the bursting patterns of Figure 3 are displayed in Figure 8 and Figure 9, which show a group of typical bursting patterns. In particular, they exhibit a large amount of complex bursting patterns showing compound structures, characterized by multiple clusters of small-amplitude value that can be observed in each cycle of bursting.

**Figure 10:**
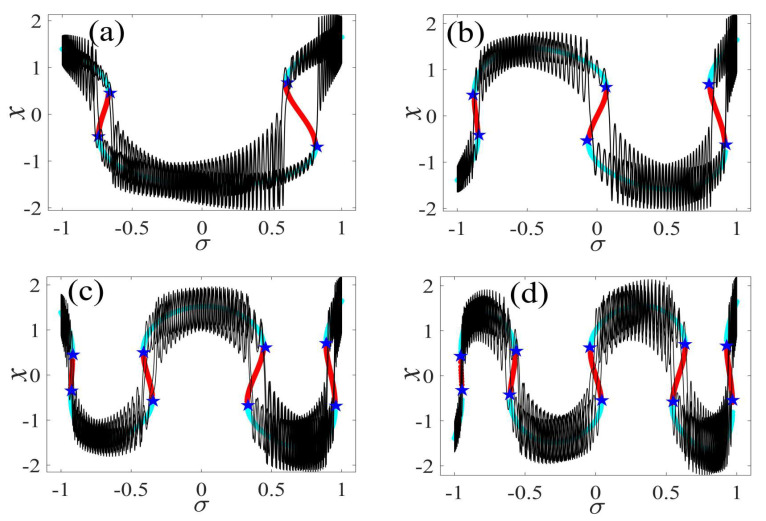
Transformed phase diagram for (**a**) Figure 5a, (**b**) Figure 6a, (**c**) Figure 7a, and (**d**) Figure 8a. (The solid cyan lines represent the stable values, the red lines represent the unstable values, the blue pentagram represents the fold bifurcation points, the black lines represent the time series within a cycle).

**Figure 11:**
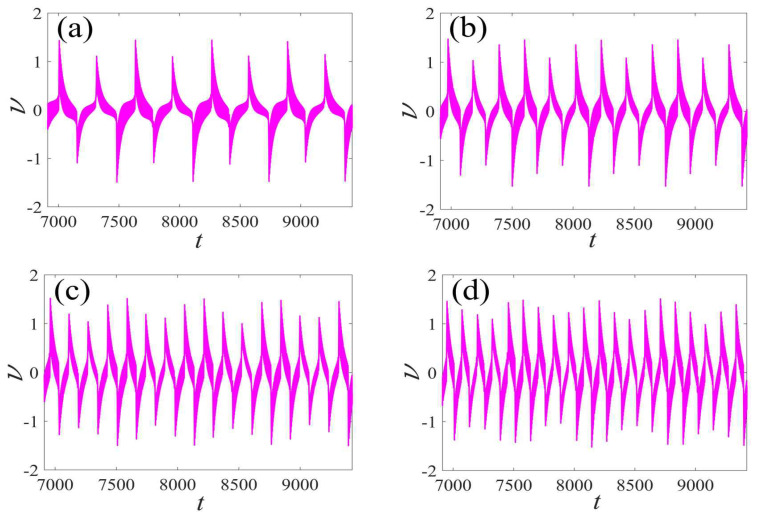
Bursting patterns of voltage for (**a**) Figure 5a, (**b**) Figure 6a, (**c**) Figure 7a, and (**d**) Figure 8a. (The magenta lines represent time series).

Furthermore, the bursting responses of an externally and parametrically excited post-buckled beam harvester with two slow commensurate excitation frequencies are calculated by integrating Equations (8) and (9), and the associated transformed trajectories are overlayed with the equilibrium point curves, as shown in Figure 10. It can be seen that the transformed trajectories fit nicely with the bifurcation diagrams. Simultaneously, the jump phenomenon and hysteresis behavior are achieved. In addition, the corresponding time series are also recorded in Figure 11. When the time is less than eleven cycles (about 7000 s), the transient results are thrown away, and only the steady-state results are retained. From Figure 11, we can see that as the frequency $\omega_{1}$ for a fixed $\omega_{1}$ grows, the number of large response burstings in the same period increases, and a high efficiency of vibration in the same period is desired by energy harvesting technology.

## 4. Performance Comparison of the Fundamental and the Multi-Frequency Slow Excitations

In the above section, we analyzed the bursting mechanism of an externally and parametrically excited energy harvester, and then, we compared the harvesting performance of the fundamental and the double frequency slow excitations. To compare the power output of the system, the average voltage in a period is expressed as

(13) $$\nu =\frac{1}{T}\int_{0}^{T}\nu dt$$

and the power is described as

(14) $$P=\chi \nu^{2}$$

where the period $T=2\pi /\omega_{0}$.

### 4.1. Response of the Fundamental and the Multi-Frequency External Excitations

**Figure 12:**
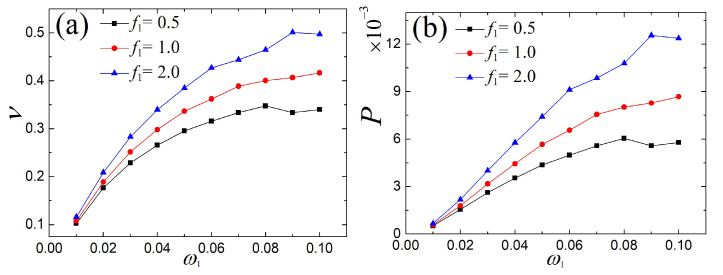
Comparison of (**a**) voltage and (**b**) power of the fundamental and the multi-frequency external excitations.

**Figure 13:**
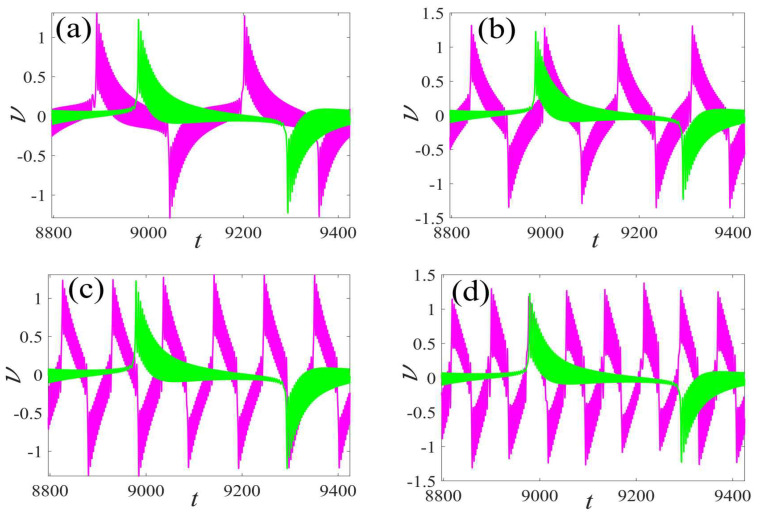
Comparison of the voltage waveform of different external excitation frequencies in a period: (**a**) $\omega_{1}=0.02$ vs. $\omega_{0}$, (**b**) $\omega_{1}=0.04$ vs. $\omega_{0}$, (**c**) $\omega_{1}=0.06$ vs. $\omega_{0}$, and (**d**) $\omega_{1}=0.08$ vs. $\omega_{0}$. (The magenta curve represents response of multi-frequency and the green describes response of fundamental frequency).

In this subsection, we start by discussing the output electric response of the single- and the multi-frequency external excitations. The voltage and power under three different amplitudes of external excitation are given in Figure 12, where it can be seen that with the growth of the double frequency ratio, the output voltage and power rapidly increase, except that they fluctuate a little at individual frequencies. Furthermore, the corresponding time series of voltage for certain double frequencies are plotted in Figure 13. It was found that the fast-paced voltage responses are depicted as the multiple double-frequency excitations increase. Obviously, the fast-paced response is preferred for larger electrical capture.

### 4.2. Response of the Single and the Multi-Frequency Parametric Excitations

**Figure 14:**
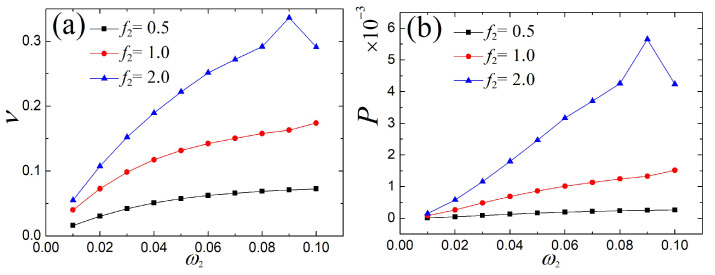
Comparison of (**a**) voltage and (**b**) of the single- and double-frequency parametric excitations.

**Figure 15:**
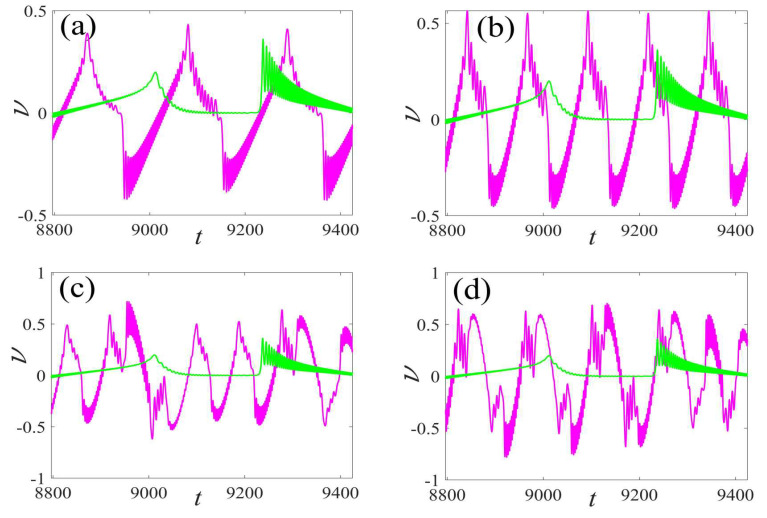
Comparison of the voltage waveform of different parametric excitation frequencies in a period: (**a**) $\omega_{2}=0.03$ vs. $\omega_{0}$, (**b**) $\omega_{2}=0.05$ vs. $\omega_{0}$, (**c**) $\omega_{2}=0.07$ vs. $\omega_{0}$, and (**d**) $\omega_{2}=0.09$ vs. $\omega_{0}$. (The magenta curve represents response of multi-frequency and the green describes response of fundamental frequency).

In this section, the performance comparison of the single and the multi-frequency parametric excitations are described, as shown in Figure 14. As the frequency $\omega_{2}$ grows, the output voltage and power rapidly increase, except for the double frequency $\omega_{2}=0.10$. The time series of voltage for certain double frequencies are displayed in Figure 15. Similarly, the fast-paced voltage responses are observed.

### 4.3. Response of the Fundamental and the Multi-Frequency External and Parametric Excitations

**Figure 16:**
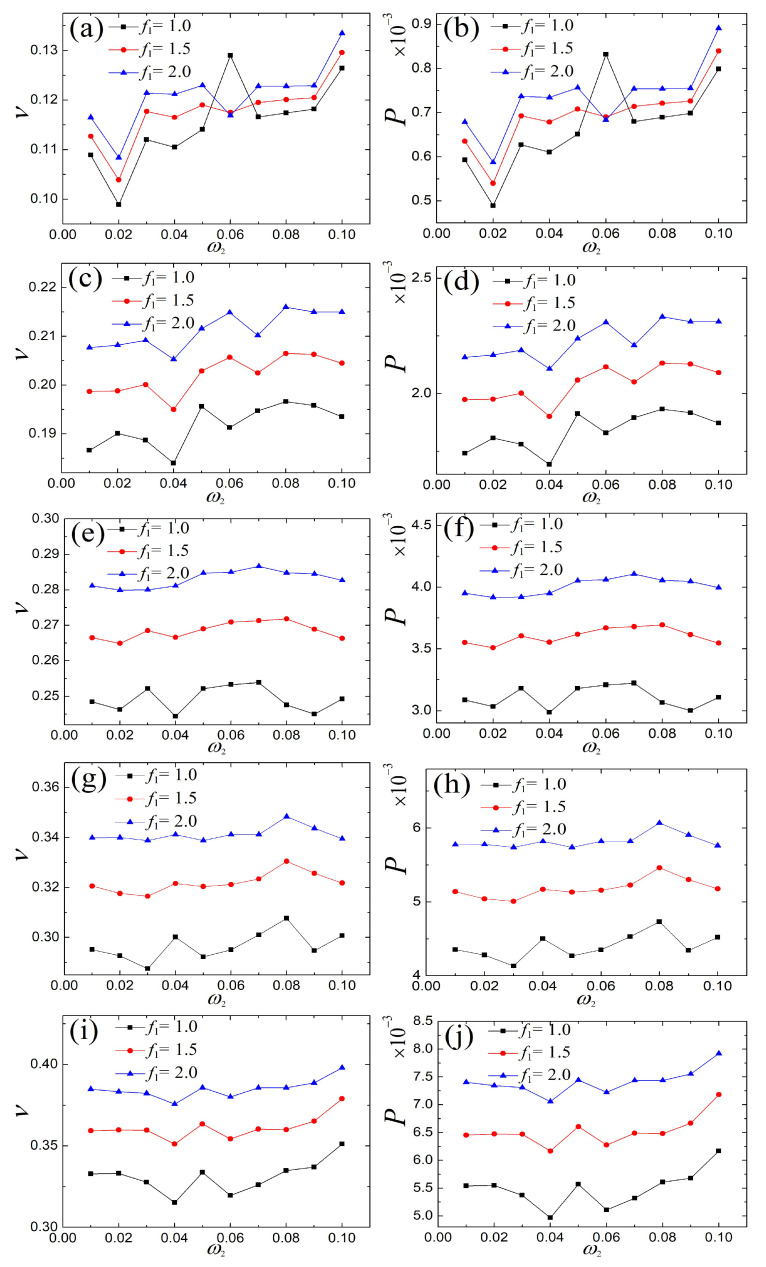
Comparison of the voltage and power of the fundamental and the double-frequency external and parametric excitations: (**a**,**b**) $\omega_{1}=0.01$, (**c**,**d**) $\omega_{1}=0.02$, (**e**,**f**) $\omega_{1}=0.03$, (**g**,**h**) $\omega_{1}=0.04$, and (**i**,**j**) $\omega_{1}=0.05$.

**Figure 17:**
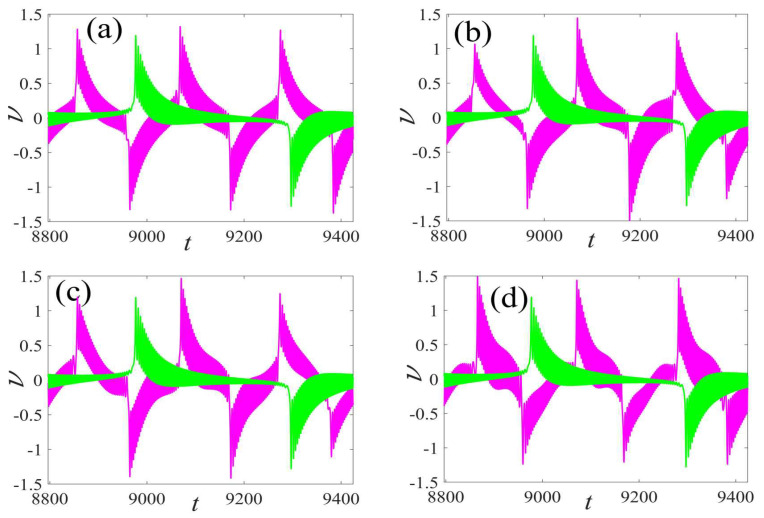
Comparison of the voltage waveform of Figure 16e for different excitation frequencies in a period: (**a**) $\omega_{2}=0.03$ vs. $\omega_{0}$, (**b**) $\omega_{2}=0.05$ vs. $\omega_{0}$, (**c**) $\omega_{2}=0.07$ vs. $\omega_{0}$, and (**d**) $\omega_{2}=0.09$ vs. $\omega_{0}$. (The magenta curve represents response of multi-frequency and the green describes response of fundamental frequency).

In carrying out the same process as the above section, the corresponding performance comparison of the single- and the multi-frequency external and parametric excitations is plotted in Figure 15. It is observed from Figure 16 that large average voltages and power can be obtained under the multi-frequency external and parametric excitations, and the associated voltage waveform is overlayed with the fundamental frequency excitation in Figure 17, which further demonstrates the high efficiency of the multi-frequency external and parametric slowly varying excitations.

## 5. Verification of the Proposed Results

**Figure 18:**
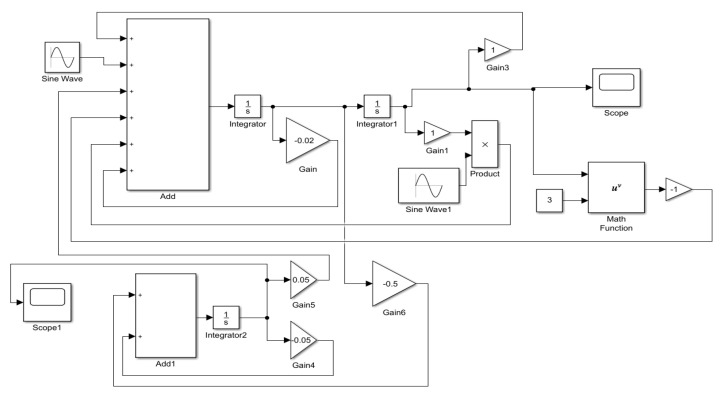
Simulink model of an externally and parametrically excited buckled beam harvesting system.

To verify the proposed results, we report another method to calculate the numerical solutions based on Simulink. The Simulink simulations were carried out for systems (8) and (9). The Simulink model of the systems is plotted in Figure 18, where the system of the block diagram Add represents the mechanical Equation (8), and the block diagram Add1 describes the circuit Equation (9). Furthermore, electromechanical coupling is achieved with gains 5 and 6, the external base excitation is implemented by adding Sine Wave, and the parameter excitation is accomplished by multiplying Sine Wave1. The response output is realized by Scope and Scope1 under default system settings.

**Figure 19:**
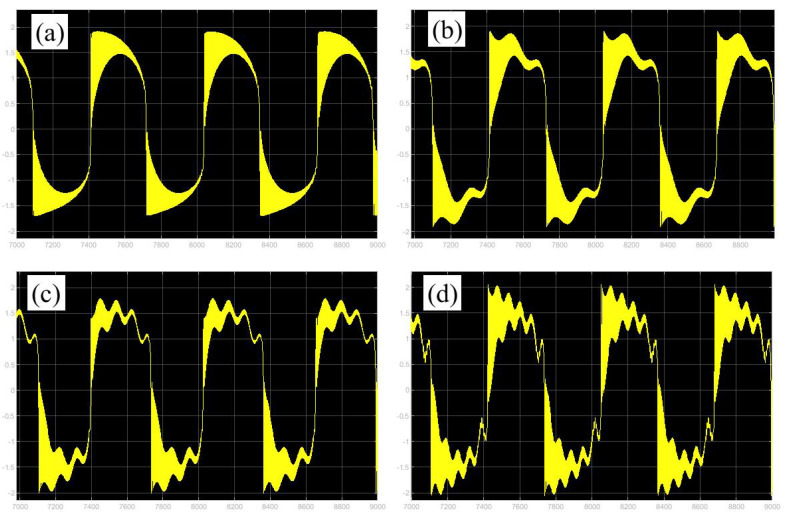
Validation of bursting patterns of displacement for Figure 8. (**a**) corresponding to Figure 8a, (**b**) corresponding to Figure 8b, (**c**) corresponding to Figure 8c, (**d**) corresponding to Figure 8d.

**Figure 20:**
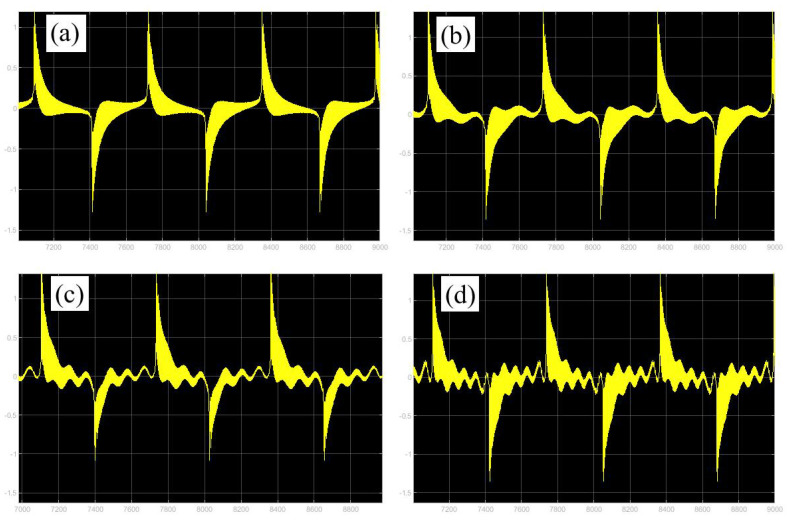
Validation of bursting patterns of displacement for Figure 9. (**a**) corresponding to Figure 9a, (**b**) corresponding to Figure 9b, (**c**) corresponding to Figure 9c, (**d**) corresponding to Figure 9d.

Moreover, the corresponding time histories of the displacement response and the voltage of Figure 8 and Figure 9 are given in Figure 19 and Figure 20. By comparing the two different methods, one can find excellent agreement. Therefore, the proposed method is capable of accurately predicting the dynamic response to evaluate the energy harvesting efficiency of the externally and parametrically excited buckled beam harvester.

## 6. Conclusions

This paper exploited the bursting response of a buckled beam harvester under combined external and parametrical slow excitations. The bursting mechanisms of the system were addressed. The one-parameter analyses of equilibria points were presented, and the multi-valued characteristic, two jump phenomenons, and hysteresis behavior were obtained. Bifurcation diagrams of many patterns were detected. The harvesting performance of the fundamental and the two slow commensurate excitation frequencies were compared. This paper obtained the following meaningful conclusions:

(1) The static equilibrium curve had three solutions when σ lay between two-fold bifurcations, and two jump phenomenons and hysteresis behavior were observed. In addition, for the system with two slow commensurate excitation frequencies, the number of unstable solution intervals on the one-parameter bifurcation curve was equal to the frequency ratio $\omega_{1}$ to $\omega_{0}$, and the number of folded bifurcation points was twice the frequency ratio.
(2) The qualitative bursting behaviors of the system were detected by using the voltage waveform, and the bifurcation of the equilibrium curves was verified via the transformed phase trajectory. In addition, the proposed bursting results were also checked by employing the Simulink.
(3) As the double frequencies grew, the fast-paced voltage responses in a period increased, which caused an increase in the average voltage and power, and fast-paced vibration was desired by the energy-harvesting technology.

## Data Availability

The data presented in this study are available on request from the corresponding author.
